# Impaired response inhibition to negative emotional stimuli in depressed adolescents with non-suicidal self-injury: a neurophysiological perspective

**DOI:** 10.3389/fpsyt.2025.1559068

**Published:** 2025-04-01

**Authors:** Lin Zhao, Su Hong, Xinyu Peng, Xiaoqing He, Jinhui Hu, Lingli Ma, Xinyi Liu, Wanqing Tao, Ran Chen, Zhenghao Jiang, Chenyu Zhang, Jing Liao, Jiaojiao Xiang, Qi Zeng, Linqi Dai, Qi Zhang, Wo Wang, Li Kuang

**Affiliations:** ^1^ Department of Psychiatry, The First Affiliated Hospital of Chongqing Medical University, Chongqing, China; ^2^ Mental Health Center, University-Town Hospital of Chongqing Medical University, Chongqing, China; ^3^ Psychiatric Center, The First Affiliated Hospital of Chongqing Medical University, Chongqing, China

**Keywords:** nonsuicidal self-injury, adolescents, response inhibition, negative emotional stimulation, time-frequency analysis

## Abstract

**Background:**

Non-suicidal self-injury (NSSI) is an increasingly recognized clinical and public health issue among adolescents. This behavior exhibits certain addictive characteristics, leading it to be classified as a behavioral addiction. Response inhibition is believed to play a role in the occurrence of addictive behaviors and is often impaired in the context of negative emotional states. In this study, we compared the behavioral performance, ERP time-domain and time-frequency characteristics among depressed adolescents with NSSI, depressed adolescents, and healthy controls when exposed to negative emotional stimuli. The aim was to investigate the impact of negative emotional stimuli on the response inhibition in depressed adolescents with NSSI, clarify the role of response inhibition in NSSI behaviors, and provide neurophysiological evidence for its underlying mechanisms.

**Methods:**

Seventy-one depressed adolescents with NSSI (MDD+NSSI group: 12 males, 59 females; mean age: 14.37 years), 55 depressed adolescents (MDD group: 24 males, 31 females; mean age: 15.29 years) and 25 healthy subjects (HC group, 13 males, 12 females, mean age: 15.72 years) were recruited to perform a two-choice oddball task related to negative emotional cues. All participants completed a self-administered questionnaire to gather demographic information. A trained psychiatrist administered the Hamilton Depression Scale (HAMD-17) to assess depression severity and used the Ottawa Self-Injury Inventory (OSI) to assess self-injury. Multichannel EEG was recorded continuously from 64 scalp electrodes using the Curry 8 system. EEG signal preprocessing and analysis was performed offline using the EEGLAB toolbox in MATLAB. The ERP time-domain features related to response inhibition were extracted from the difference waves, converted to the time-frequency features using the short-time Fourier transform (STFT), and the time-frequency values of the region of interest (ROI) were extracted and statistically analyzed.

**Results:**

Under exposure to negative emotional stimuli, depressed adolescents with NSSI exhibited significantly larger P300 amplitudes compared to both depressed adolescents and healthy controls. Moreover, depressed adolescents with NSSI showed significant event-related synchronization (ERS) in the Delta and Theta bands of FCz electrode from 0 to 0.6 seconds, and event-related desynchronization (ERD) in the Theta and Alpha bands of Pz electrode from 0.2 to 1.2 seconds, collectively reflecting functional processes associated with response inhibition.

**Conclusions:**

Depressed adolescents with NSSI showed increased P3d amplitudes, enhanced Delta/Theta ERS, and heightened Theta/Alpha ERD when receiving negative emotional stimuli, suggesting that depressed adolescents with NSSI have impaired response inhibition, which may contribute to the development of NSSI.

## 1 Introduction

Non-suicidal self-injury (NSSI) is an increasingly recognized clinical and public health issue predominantly observed in adolescence and early adulthood (1). NSSI refers to deliberate and direct self-inflicted bodily harm without suicidal intent, conducted in ways that are not socially or culturally sanctioned, including behaviors such as scratching, hitting, burning, cutting, self-mutilation, and interfering with wound healing (2, 3). This behavior is highly hazardous, marked by repetitiveness, intentionality, and lack of control. While NSSI lacks suicidal intent and is not fatal in isolated episodes, its recurrence is a major trigger for suicidal behaviors, including attempts and completions, and a strong predictor of suicide risk (4, 5). Studies show that each additional occurrence of NSSI increases the likelihood of future suicide attempts by sevenfold (6). Previous studies have shown that adolescents often experience negative emotions such as depression, anxiety, and anger before engaging in NSSI (7). This behavior is frequently employed as a maladaptive coping strategy in response to distress or negative emotions, evolving from occasional episodes to habitual patterns that are difficult to resolve independently (8). NSSI exhibits both threatening and relieving characteristics, often preceded by a pronounced sense of urgency or craving and followed by rapid alleviation of discomfort, accompanied by transient feelings of euphoria (9). This reinforcing cycle contributes to its recurrence (10). This pattern of NSSI behavior parallels that of substance addiction, prompting some researchers to classify it as a behavioral addiction with a comparable neurobiological foundation (11, 12).

Previous studies have identified deficits in response inhibition among individuals with substance addiction (13, 14), which may predate substance use and contribute to addiction development (15, 16). Response inhibition refers to the ability to suppress inappropriate dominant behaviors, allowing for more flexible and goal-directed responses to external stimuli (17, 18). Stronger dominant responses increase the difficulty of inhibition (19). Research has shown that response inhibition is impaired in negative emotional contexts (20), potentially leading to impulsive behaviors, including self-harm, aggression, suicide risk, social hostility, gambling, risky sexual behavior, and substance abuse (21). Neuroimaging evidence further indicates prefrontal cortex dysfunction and impaired inhibitory control in individuals with NSSI (22).

Previous studies have widely used neuroelectrophysiological techniques to examine brain activity related to response inhibition, often employing tasks such as the Go/NoGo task, Stop-signal task, and two-choice Oddball task (23). The two-choice Oddball task is preferred for its ability to assess both reaction time and accuracy. In this task, participants respond to frequent standard stimuli while suppressing habitual responses to correctly react to infrequent deviant stimuli, reflecting the strength of response inhibition (24). Event-related potential (ERP) components, N2 and P3, are closely associated with response inhibition (25–27). The N2 component is a negative deflection occurring approximately 200 ms after stimulus presentation, reflecting early-stage conflict monitoring and cognitive control (28). Studies have shown delayed N2 latency in frontal electrodes following mental fatigue, indicating a reduction in the speed of early conflict monitoring during response inhibition (29). The P3 component is a positive deflection observed around 300 ms post-stimulus, representing late-stage top-down inhibitory mechanisms to resolve conflict and allocate cognitive resources to stimuli (30). Larger N2 and P3 amplitudes are indicative of greater cognitive resources allocated to response inhibition, while longer latencies suggest slower cognitive processing (31, 32). Studies have shown that heavy drinkers exhibit prolonged P3 latencies and increased N2 and P3 amplitudes under alcohol-related cues compared to neutral cues, indicating impaired inhibitory control (33). Similarly, our previous work found that depressed adolescents with NSSI exhibit deficits in inhibitory control when exposed to self-harm cues, evidenced by increased P3 difference wave (P3d) amplitudes (34).

In addition to examining the temporal characteristics of EEG activity associated with response inhibition, it is equally important to investigate its time-frequency features. Neural responses to stimuli involve not only event-related potentials (ERPs) but also modulations in transient neural oscillations, reflected as increases or decreases in specific frequency band power, referred to as event-related synchronization (ERS) and event-related desynchronization (ERD), respectively (35). Time-frequency analysis, which examines EEG power changes within specific temporal and frequency ranges, provides a complementary approach to traditional ERP analysis. Compared to standard temporal-domain ERP analysis, time-frequency analysis offers a unique advantage in uncovering information that cannot be accessed through temporal methods alone. Previous studies suggest that N2 and P3 components alone may not sufficiently capture the complexity of response inhibition processes. Time-frequency analyses of response inhibition have shown increased frontal theta and central delta activity, which are thought to be associated with response inhibition. Theta and delta activities are considered overlapping but distinct functional processes embedded within common ERP components such as N2 and P3 (36, 37). Specifically, increased theta activity in response to NoGo stimuli reflects the initial detection of conflict between response execution and inhibition, analogous to the NoGo-N2 component (36). In contrast, increased delta activity in response to NoGo stimuli reflects both motor inhibition (similar to NoGo-P3) (38) and the motivational relevance and salience of target stimuli (39, 40). Therefore, time-frequency analysis provides critical insights into oscillatory dynamics, offering a valuable perspective for understanding the neural mechanisms underlying response inhibition.

Based on these considerations, this study aims to examine whether response inhibition is impaired in depressed adolescents with NSSI under negative emotional stimuli. Using a two-choice Oddball paradigm with neutral and negative emotional cues, we compared behavioral performance, ERP temporal characteristics, and time-frequency features among adolescents with NSSI and depression, those with depression alone, and healthy controls. These findings aim to clarify the role of response inhibition in NSSI behaviors and provide neuroelectrophysiological evidence for its underlying mechanisms.

## 2 Methods

### 2.1 Participants

The Academic Ethics Committee of the First Affiliated Hospital of Chongqing Medical University approved this study (approval number: 2021-546). All participants and their legal guardians were fully informed about the study’s procedures and objectives and signed written informed consent forms prior to participation. The study comprised 25 healthy participants (healthy control group: 13 males, 12 females; mean age: 15.72 years), 55 depressed adolescents (MDD group: 24 males, 31 females; mean age: 15.29 years), and 71 depressed adolescents with NSSI (MDD+NSSI group: 12 males, 59 females; mean age: 14.37 years). All depressed adolescents in this study were experiencing their first episode and had not received prior treatment. Participants were recruited from outpatient and inpatient wards at the First Affiliated Hospital and University Town Hospital of Chongqing Medical University. Before participation, all subjects underwent screening and assessment by two senior psychiatrists using the MINI-International Neuropsychiatric Interview (M.I.N.I. KID 5.0) (41), supplemented by scale evaluations for diagnosis. All patients met the ICD-11 criteria for a diagnosis of major depressive disorder (MDD) (42). The criteria for NSSI adhered to the Diagnostic and Statistical Manual of Mental Disorders, 5th Edition (DSM-5) (43). During the same time frame, we also publicly advertised recruitment on the Internet for healthy individuals who were age- and sex-matched to the patients as the healthy control group. All participants were right-handed with normal or corrected vision and hearing. Exclusion criteria encompassed a history of neurological or psychiatric disorders other than depression, chronic drug use, learning disabilities, or head injuries causing loss of consciousness.

### 2.2 General demographic data collection and clinical scale assessment

Demographic information, including age, gender, and education level, was obtained from all participants using a self-designed questionnaire. Clinical assessments were conducted by two attending psychiatrists or higher-level specialists using the Hamilton Depression Rating Scale (HAMD) (44) and the Ottawa Self-Injury Inventory (OSI) (45). The HAMD was used to evaluate the severity of depression, while the OSI was employed to assess specific details of NSSI behaviors. Details are shown in **Table 1**.

**Table 1:** Demographic and clinical characteristics of participants in the HC group, MDD group, and MDD+NSSI group.

	HC group (n=25) *M (SD)*	MDD group (n=55) *M (SD)*	MDD+NSSI group (n=71) *M (SD)*	*F/χ2*	*P*
Age (years)	15.72(2.031)	15.29(1.536)	14.37(1.597)	8.286	<0.001
Sex (male/female)	13/12	24/31	12/59	15.329	<0.001
HAMD score	1.24(1.832)	22.55(3.248)	23.44(4.275)	381.397	<0.001
NSSI number	/	/	10.24 ± 3.911		
NSSI first onset age	/	/	12.80 ± 1.696		
NSSI type					
Cutting			71/71		
Pinching			13/71		
Biting			9/71		
Knocking			10/71		
Burning			1/71		

HC, healthy controls; MDD, major depressive disorder; MDD + NSSI, MDD with nonsuicidal self-injury; HAMD, Hamilton Depression Scale.

### 2.3 Stimulus task and procedure

#### 2.3.1 Stimuli

This study utilized emotional facial images sourced from the Chinese Facial Affective Picture System (CFAPS) (46). The stimuli included two types of emotional face pictures: neutral (7 pictures) and negative (6 pictures). In the neutral cue block, one neutral face picture was designated as the standard stimuli, while six other neutral face pictures served as deviant stimuli. In the negative cue block, the same neutral face picture was used as the standard stimuli, and six negative face pictures served as deviant stimuli. All pictures were randomly chosen by the experimenter from the CFAPS system, matched for valence and arousal, and standardized to identical dimensions (260 × 300 pixels; 100 pixels per inch).

#### 2.3.2 Procedure

**Figure 1:**
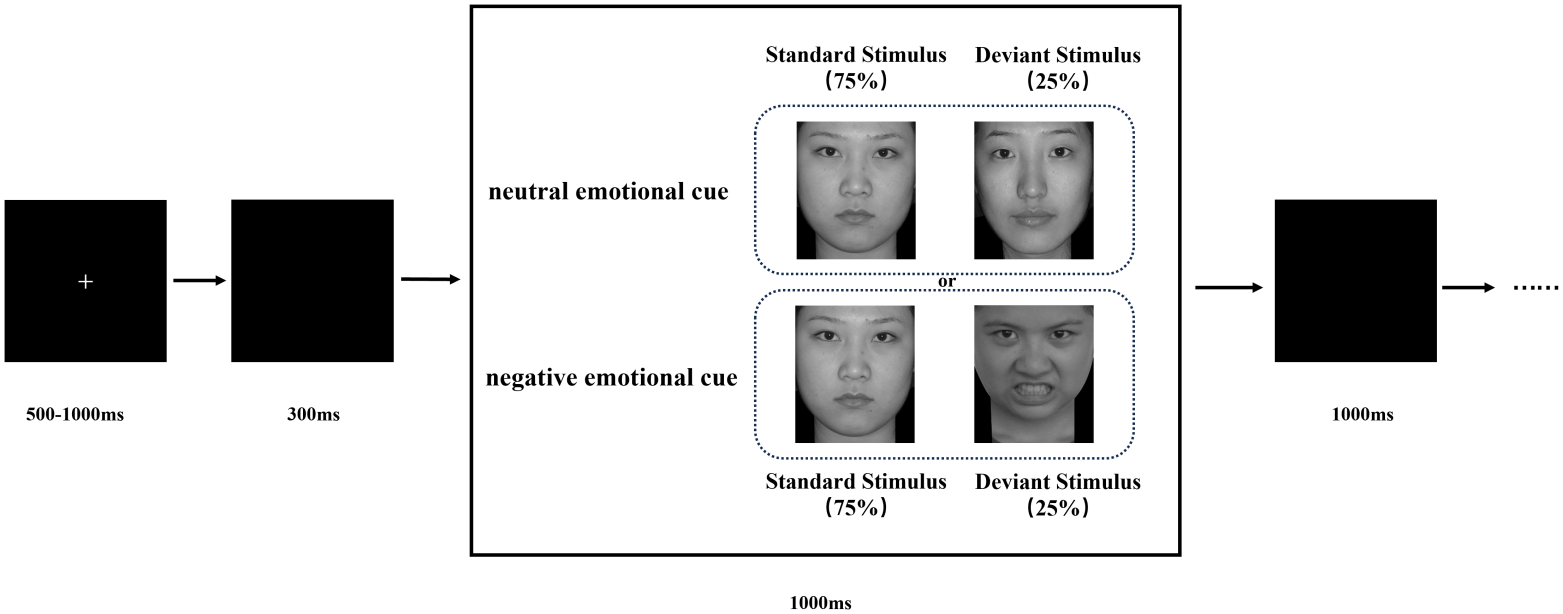
Schematic illustration of the experimental procedure and stimulus examples. Each trial is presented a single deviant or standard stimulus. Subjects pressed the “1” key and “2” key in response to standard and deviant stimuli, respectively.

Participants were seated in a quiet room, approximately 60 cm from a computer screen, with their bodies relaxed and heads as stationary as possible. The stimuli were presented on the computer screen using E-Prime 3.0 software. The stimulus task paradigm, illustrated in **Figure 1**, consisted of two blocks: a neutral emotional cue block and a negative emotional cue block. Each block comprised 200 stimulus presentations, with participants given a rest period between blocks. In the neutral cue block, standard stimuli (a neutral face picture) appeared 150 times (75%), and deviant stimuli (six other neutral face pictures appearing randomly) appeared 50 times (25%). Similarly, in the negative cue block, standard stimuli (the same neutral face picture) appeared 150 times (75%), while deviant stimuli (six other negative face pictures appearing randomly) appeared 50 times (25%). Each stimulus presentation began with a fixation cross displayed for 500-1000 ms, followed by a 300 ms blank screen. The stimulus image then appeared randomly on the screen for up to 1000 ms, or until the participant responded. Following each stimulus, a blank screen was displayed for 1000 ms before the next stimulus cycle began. Participants were instructed to respond as quickly and accurately as possible to the stimuli by pressing the corresponding button. They pressed the ‘1’ key for standard stimuli and the ‘2’ key for deviant stimuli. Before the main experiment, participants completed a practice session, with the experiment commencing only after achieving at least 80% accuracy. The experiment lasted approximately 30 minutes.

### 2.4 EEG acquisition and preprocessing

EEG data were acquired using a 64-channel Neuroscan Quick cap and recorded with the Curry 8 system. Electrodes were positioned according to the international 10/20 system to ensure accurate signal acquisition. Vertical eye electrodes (VEOG) were placed above and below the left eye, and horizontal eye electrodes (HEOG) were positioned on the outer canthi of both eyes to monitor eye movements and remove associated artifacts. The reference electrode was placed between Cz and CPz. EEG signals were recorded at a sampling rate of 1000 Hz. Electrode impedance was minimized using conductive paste, and data acquisition commenced only when all impedances were below 5 kΩ.

EEG signal processing and offline analysis were conducted in MATLAB using the EEGLAB toolbox (47). EEG data were resampled to 500 Hz and filtered using a 0.1-30 Hz band-pass filter and a 48-52 Hz notch filter to remove power frequency interference. Unused electrodes (e.g., EKG, EMG, CB1, and CB2) were excluded. EEG data were segmented into 1200 ms epochs, including 200 ms pre-stimulus and 1000 ms post-stimulus intervals. Only epochs with correct responses (containing at least 20 correct response markers) were retained, epochs with incorrect responses or no response, and epochs containing significant artifacts were excluded, and any bad electrodes were corrected using spherical interpolation when necessary. Independent component analysis (ICA) was applied to remove artifacts, including blinks, horizontal eye movements, and muscle-related noise (48). EEG segments with correct responses to the two emotional stimulus conditions were selected and averaged for each subject to analyze responses to emotional stimuli. Baseline correction was applied by subtracting the 200 ms pre-stimulus baseline from the post-stimulus waveform.

### 2.5 ERP analysis

#### 2.5.1 Time domain (ERP) analysis: N2 and P3

ERP difference waveforms were calculated by subtracting standard stimuli from deviant stimuli for neutral and negative emotional face conditions. Following prior research (49), ERP analysis focused on the peak latency and average amplitude, defined as the mean amplitude within ±10 ms of the peak ERP component across specific electrode groups during designated time windows. Visual inspection of ERP waveforms at central electrodes (Fz, FCz, Cz, CPz, Pz) revealed that the N2 component was most prominent at FCz and the P3 component at Pz. Thus, the following time windows and electrode sites were analyzed: N2 (200–300 ms) at FCz and P3 (350–550 ms) at Pz, relative to stimulus onset.

#### 2.5.2 Time-frequency analysis

**Figure 2:**
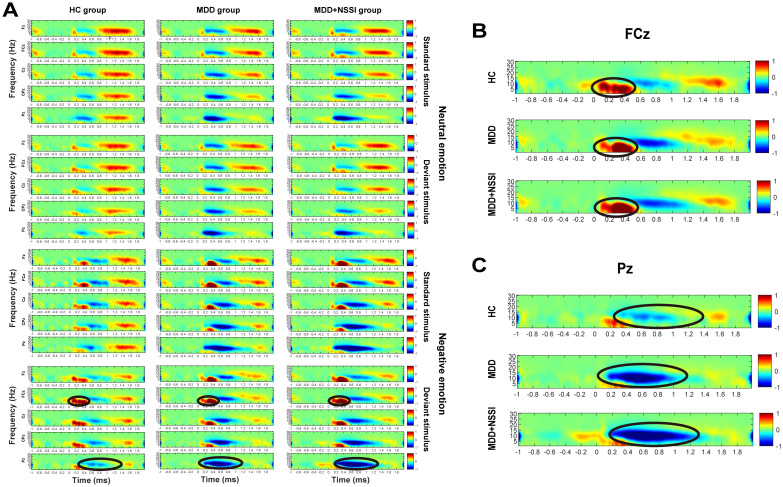
**(A)** Time-frequency energy spectra of the HC group, MDD group and MDD+NSSI group under different emotional stimulus. **(B)** Time-frequency energy spectra of the FCz electrode of the HC group, MDD group and MDD+NSSI group under negative emotional deviant stimuli. **(C)** Time-frequency energy spectra of the Pz electrode of the HC group, MDD group and MDD+NSSI group under negative emotional deviant stimuli.

A short-time Fourier transform (STFT) was applied to convert preprocessed ERP data into the time-frequency domain, allowing the extraction of power for each electrode under specific time and frequency conditions for each participant. Time-frequency power values were first calculated for each trail for each condition for each participant, and then the average time-frequency power values were calculated for all trails for each condition for each participant. A baseline correction was performed using the pre-stimulus interval from -200 to 0 ms. The time-frequency regions of interest (ROI) were identified from the distribution results (**Figure 2**) as follows: ERS at FCz (0-0.6 s) in the Delta and Theta bands, and ERD at Pz (0.2-1.2 s) in the Theta and Alpha bands. Mean values from each time-frequency ROI were extracted for statistical analysis.

### 2.6 Data analysis

Statistical analyses were conducted using SPSS Statistics 25. Baseline demographic and scale data were analyzed using one-way ANOVA and chi-square tests. A mixed ANOVA was conducted to evaluate the peak latency and mean amplitude of the N2 and P3 difference waves across the HC, MDD, and MDD+NSSI groups under different emotional stimulation conditions. Cue condition (2 levels: neutral emotional cue, negative emotional cue) was treated as a within-subject factor, while group (3 levels: HC, MDD, MDD+NSSI) was treated as a between-subject factor. A mixed ANOVA was also applied to analyze time-frequency values across the HC, MDD, and MDD+NSSI groups under different emotional stimulus conditions within each time-frequency band of interest. In this analysis, cue condition (neutral emotional cue, negative emotional cue) and stimulus type (standard, deviant) were treated as within-subject factors, while group (HC, MDD, MDD+NSSI) served as a between-subject factor. Significant interaction effects were explored further through simple effect analysis. *Post-hoc* comparisons for significant main or group effects were conducted using the Bonferroni-Holm method. The Greenhouse-Geisser correction was applied to adjust for violations of the sphericity assumption. A significance threshold of *p* < 0.05 was used for all statistical tests.

## 3 Results

### 3.1 General demographic data and clinical scale data

One-way ANOVA and chi-square tests were performed to analyze the general demographic and clinical characteristics of the three groups. The findings are presented in **Table 1**. The one-way ANOVA revealed significant differences in age among the three groups (*p* < 0.001). Chi-square test results indicated a significant difference in sex distribution across the three groups (*p* < 0.001). The predominant method of self-injury involved cutting the arms with knives or sharp objects, while less common methods included scratching, biting, burning, and hitting. The one-way ANOVA indicated significant differences in HAMD scores among the three groups (*p* < 0.001). However, no significant differences were observed between the MDD and MDD+NSSI groups (*p* > 0.05).

### 3.2 Behavioral data

**Figure 3:**
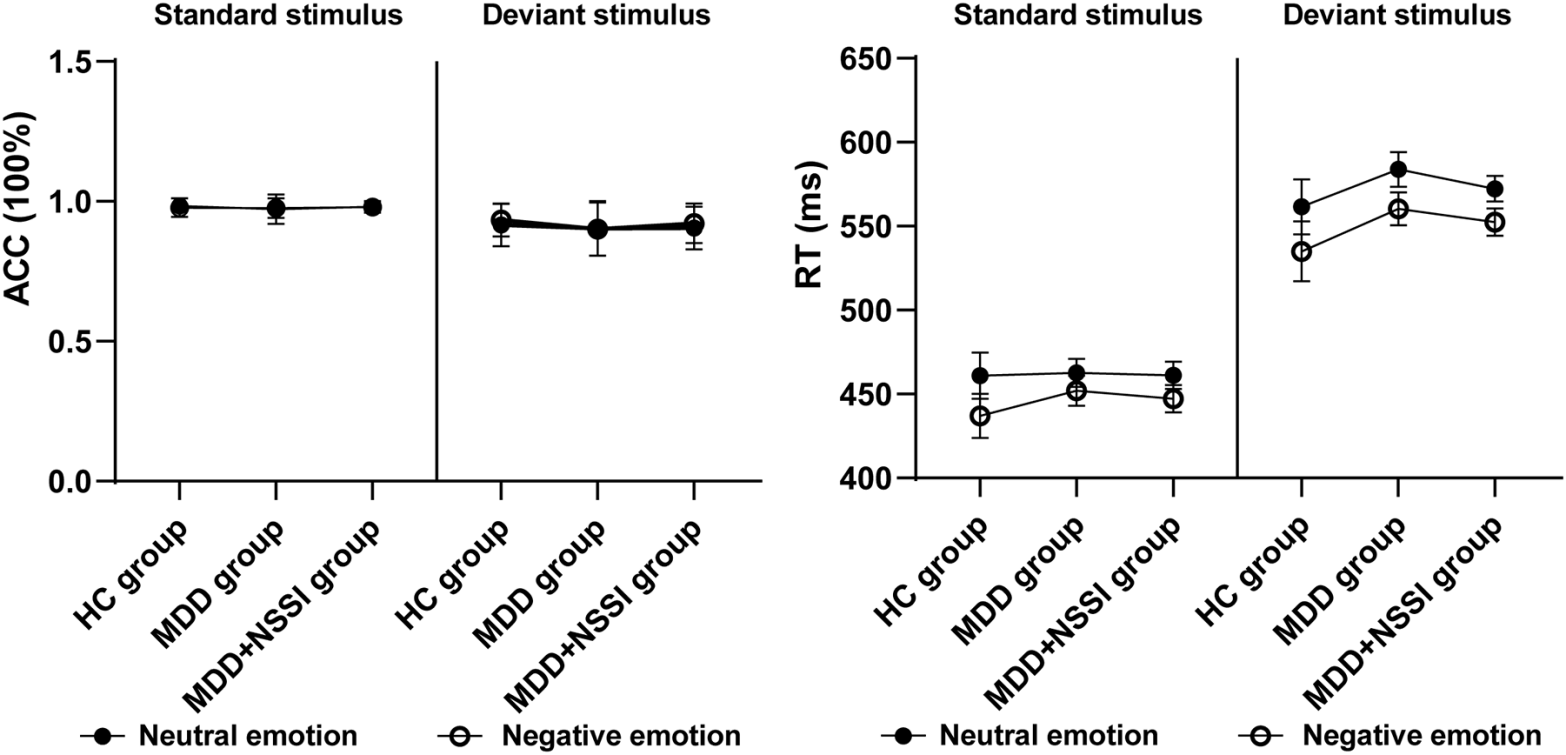
Accuracy and response time of the HC, MDD and MDD+NSSI groups under different emotional stimulus.

For ACC, ANOVA results indicated a significant main effect of stimulus [F_(1, 148)_ = 132.289, *p* < 0.001, η_p_^2^ = 0.479] and a significant interaction between emotional condition, stimulus, and group [F_(2, 148)_ = 3.673, *p* = 0.028, η_p_^2^ = 0.049]. Across all conditions, ACC was lower for deviant stimuli compared to standard stimuli. *Post hoc* simple effect analysis revealed no significant differences. For RT, significant main effects were observed for emotional condition [F_(1, 148)_ = 37.081, *p* < 0.001, η_p_^2^ = 0.205] and stimulus [F_(1, 148)_ = 688.560, *p* < 0.001, η_p_^2^ = 0.827]. RTs for negative emotions were shorter than for neutral stimuli across all participants, while RTs for deviant stimuli were longer than for standard stimuli, regardless of emotional condition. These results are summarized in **Table 2** and presented in **Figure 3**.

**Table 2:** Behavioral indicators of participants in the HC group, MDD group, and MDD+NSSI group exposed to different emotional cues.

Behavioral indicators	Emotion main effect *F(p)*	Stimulus main effect *F(p)*	Between-group main effect *F(p)*	Emotion × Stimulus interaction effect *F(p)*	Emotion × Group interaction effect *F(p)*	Stimulus × Group interaction effect *F(p)*	Emotion × Stimulus × Group interaction effect *F(p)*
ACC	2.217 (0.139)	132.289 (<0.001)	0.834 (0.436)	5.389 (0.022)	0.450 (0.638)	0.592 (0.554)	3.673 (0.028)
RT	37.081 (<0.001)	688.560 (<0.001)	0.553 (0.577)	3.417 (0.067)	0.550 (0.578)	1.012 (0.366)	0.613 (0.543)

ACC, accuracy; RT, response time.

### 3.3 EEG data

#### 3.3.1 Time-domain (ERP) data

**Figure 4:**
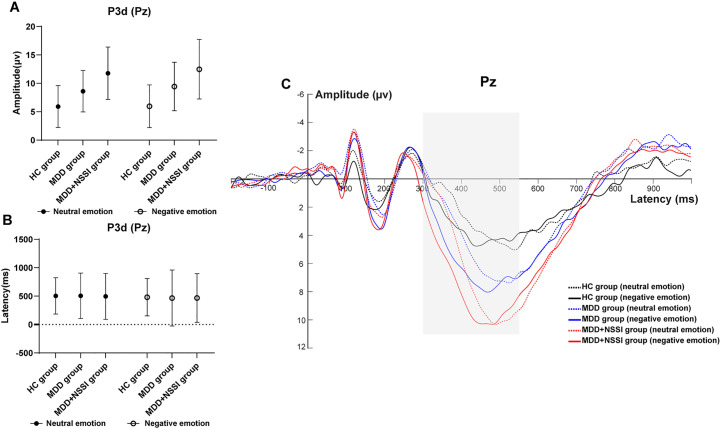
**(A)** Amplitude of P3d at Pz electrode under different emotional stimulus in HC, MDD and MDD+NSSI groups. **(B)** Latency of P3d at Pz electrode under different emotional stimulus in HC, MDD and MDD+NSSI groups. **(C)** Difference wave at Pz electrode under different emotional stimulus in HC, MDD and MDD+NSSI groups.

A repeated measures ANOVA was conducted to analyze the peak latency and mean amplitude of ERP components in the difference wave across emotional stimulation conditions. Cue condition (neutral emotional cue, negative emotional cue) served as the within-subject factor, and group (HC, MDD, MDD+NSSI) served as the between-subject factor. Due to significant group differences in age and sex, these variables were included as covariates in the repeated-measures ANOVA. The findings are summarized in **Table 3** and illustrated in **Figure 4**.

**Table 3:** P3d latency and amplitude of participants in the HC group, MDD group, and MDD+NSSI group exposed to different emotional cues on the Pz electrode.

P3d characteristics	Emotion main effect *F(p)*	Between-group main effect *F(p)*	Emotion × Group interaction effect *F(p)*
Latency(ms)	28.565 (<0.001)	0.626 (0.536)	0.729 (0.484)
Amplitude(μv)	2.935 (0.059)	23.704 (<0.001)	0.456 (0.635)

N2 Component (200–300 ms): After controlling for age and sex, no significant main effects or interactions were observed for N200 latency or amplitude.

P3 Component (300–550 ms): With age and sex as covariates, ANOVA results for the P300 component at the Pz electrode revealed a significant main effect of condition for P300 latency [F_(1, 148)_= 28.565, *p* < 0.001, η_p_^2^ = 0.162]. The main effect of condition for mean P300 amplitude approached significance [F_(1, 148)_= 2.935, *p =* 0.059, η_p_^2^ = 0.019], and the main effect of group was significant [F_(2, 148)_= 23.704, *p* < 0.001, η_p_^2^ = 0.243]. No significant differences in P300 latency were found across groups for either neutral or negative emotional cues. For P300 mean amplitude: Under neutral cues, significant differences were observed across all pairwise comparisons (*p* < 0.05). The HC group (5.911 ± 3.694) had lower amplitude than the MDD group (8.609 ± 3.648, *p* = 0.008) and the MDD+NSSI group (11.767 ± 4.617, *p* < 0.001), with the MDD group also lower than the MDD+NSSI group (*p* < 0.001). Under negative cues, similar differences were noted (*p* < 0.05). The HC group (5.964 ± 3.754) had lower amplitude than the MDD group (9.438 ± 4.271, *p* = 0.003) and the MDD+NSSI group (12.478 ± 5.242, *p* < 0.001), with the MDD group again lower than the MDD+NSSI group (*p* < 0.001). The MDD+NSSI group also showed a marginally higher P300 mean amplitude under negative cues (12.478 ± 5.242) compared to neutral cues (11.767 ± 4.617, *p* = 0.065).

#### 3.3.2 Time-frequency data

**Figure 5:**
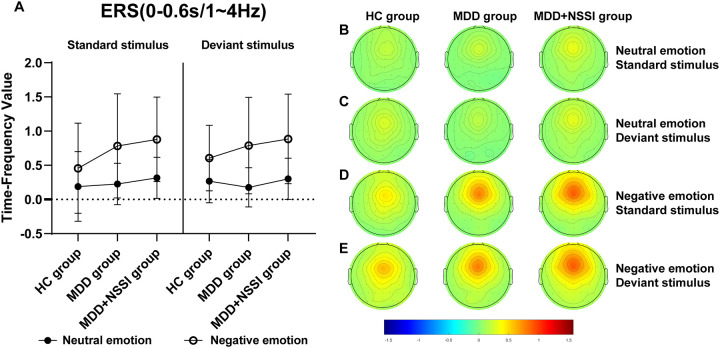
**(A)** Time-frequency values of the time-frequency band of interest (0-0.6s/1-4Hz) under different emotional stimulus in HC, MDD and MDD+NSSI groups. **(B)** Time-frequency topography of the time-frequency band of interest (0-0.6 s/1-4 Hz) in HC, MDD and MDD+NSSI groups to neutral emotional standard stimulus. **(C)** Time-frequency topography of the time-frequency band of interest (0-0.6 s/1-4 Hz) in HC, MDD and MDD+NSSI groups to neutral emotional deviant stimulus. **(D)** Time-frequency topography of the interested time frequency band (0-0.6s/1-4Hz) in HC, MDD and MDD+NSSI groups under the negative emotional standard stimulus. **(E)** Time-frequency topography of the interested time frequency band (0-0.6s/1-4Hz) in HC, MDD and MDD+NSSI groups to the negative emotional deviant stimulus.

**Figure 6:**
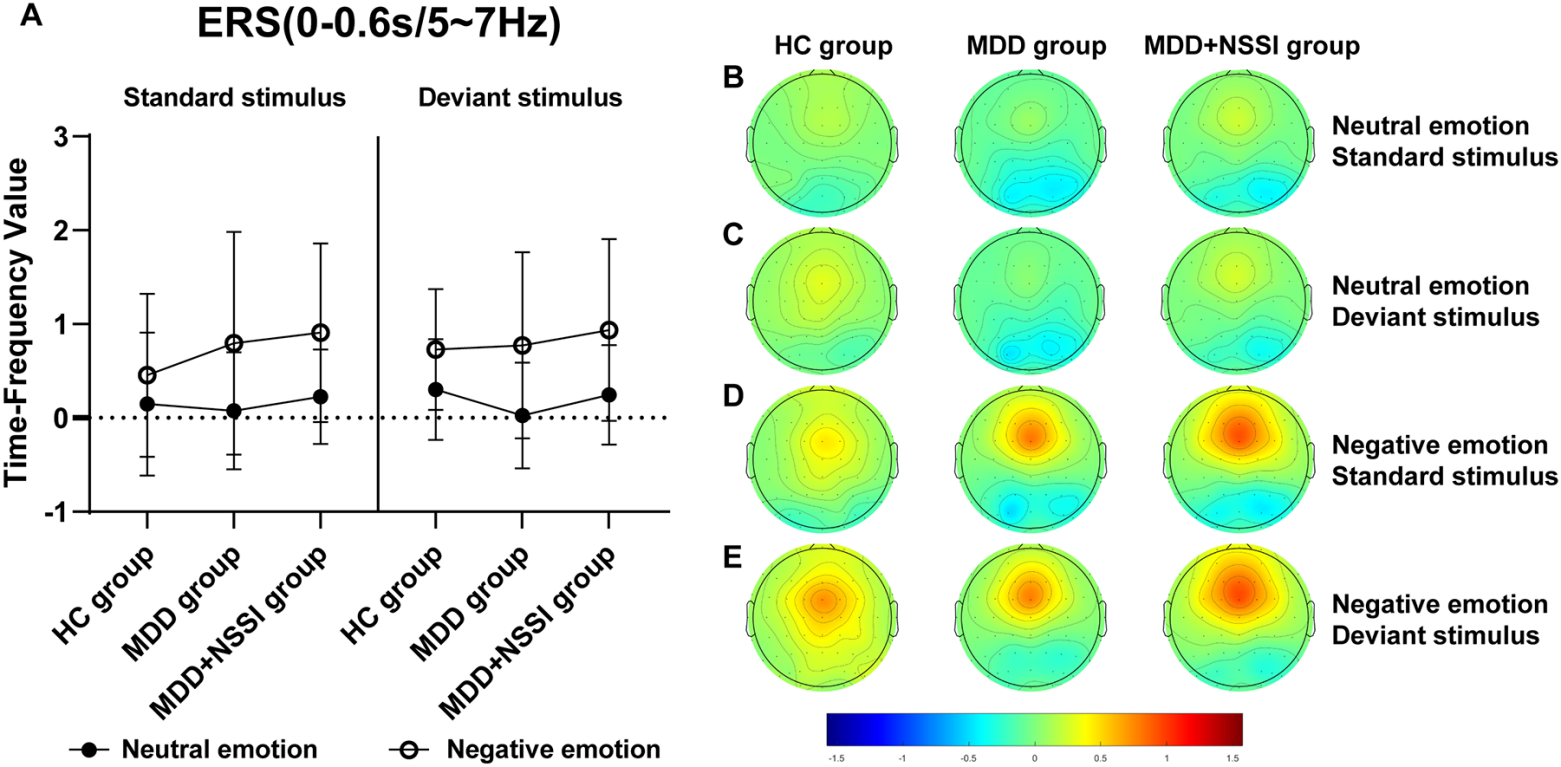
**(A)** Time-frequency values of the time-frequency band of interest (0-0.6s/5-7Hz) under different emotional stimulus in HC, MDD and MDD+NSSI groups. **(B)** Time-frequency topography of the time-frequency band of interest (0-0.6s/5-7Hz) in HC, MDD and MDD+NSSI groups to neutral emotional standard stimulus. **(C)** Time-frequency topography of the time-frequency band of interest (0-0.6s/5-7Hz) in HC, MDD and MDD+NSSI groups to neutral emotional deviant stimulus. **(D)** Time-frequency topography of the interested time frequency band (0-0.6s/5-7Hz) in HC, MDD and MDD+NSSI groups under the negative emotional standard stimulus. **(E)** Time-frequency topography of the interested time frequency band (0-0.6s/5-7Hz) in HC, MDD and MDD+NSSI groups to the negative emotional deviant stimulus.

**Figure 7:**
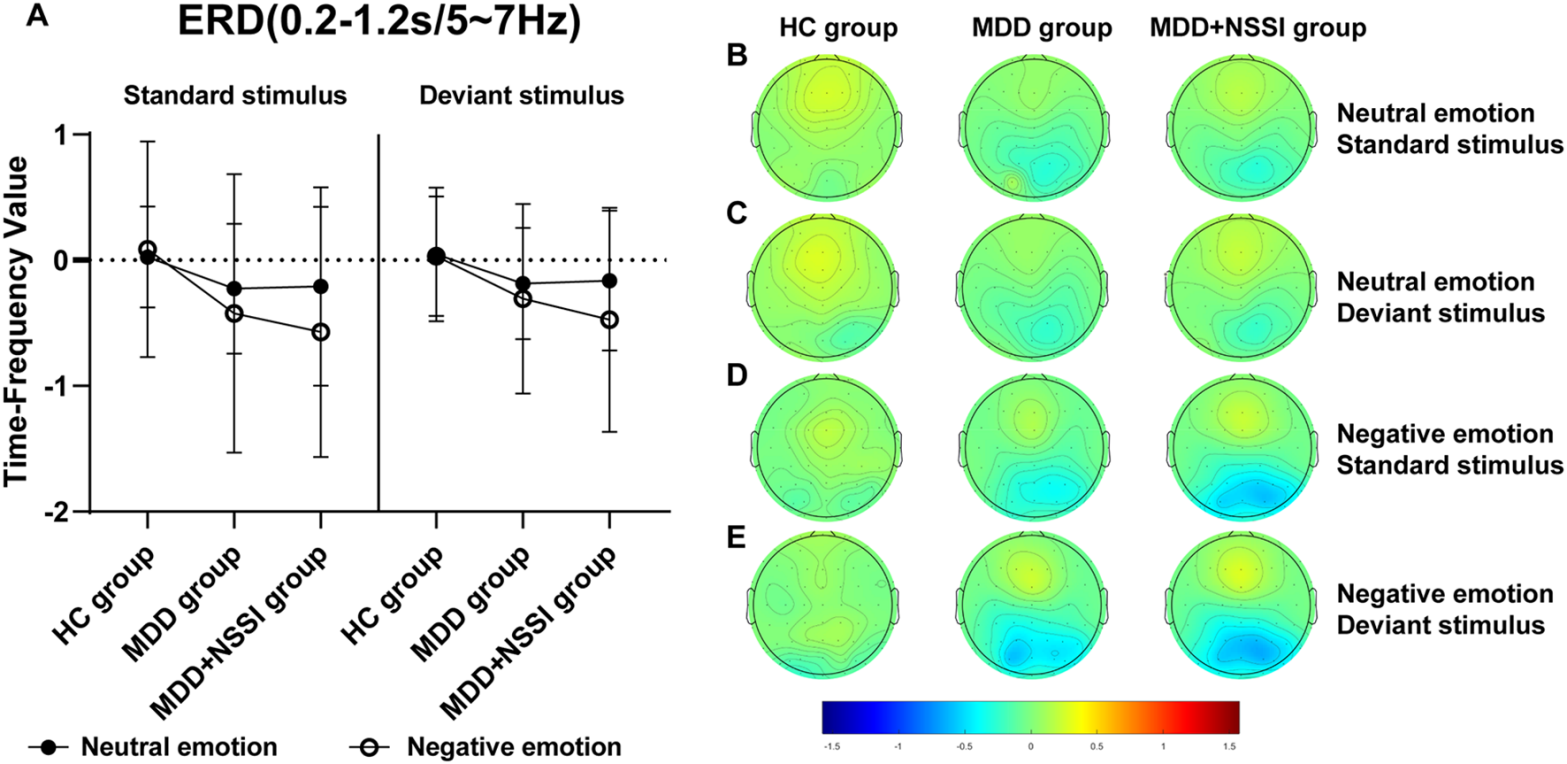
**(A)** Time-frequency values of the time-frequency band of interest (0.2-1.2s/5-7Hz) under different emotional stimulus in HC, MDD and MDD+NSSI groups. **(B)** Time-frequency topography of the time-frequency band of interest (0.2-1.2s/5-7Hz) in HC, MDD and MDD+NSSI groups to neutral emotional standard stimulus. **(C)** Time-frequency topography of the time-frequency band of interest (0.2-1.2s/5-7Hz) in HC, MDD and MDD+NSSI groups to neutral emotional deviant stimulus. **(D)** Time-frequency topography of the interested time frequency band (0.2-1.2s/5-7Hz) in HC, MDD and MDD+NSSI groups under the negative emotional standard stimulus. **(E)** Time-frequency topography of the interested time frequency band (0.2-1.2s/5-7Hz) in HC, MDD and MDD+NSSI groups to the negative emotional deviant stimulus.

**Figure 8:**
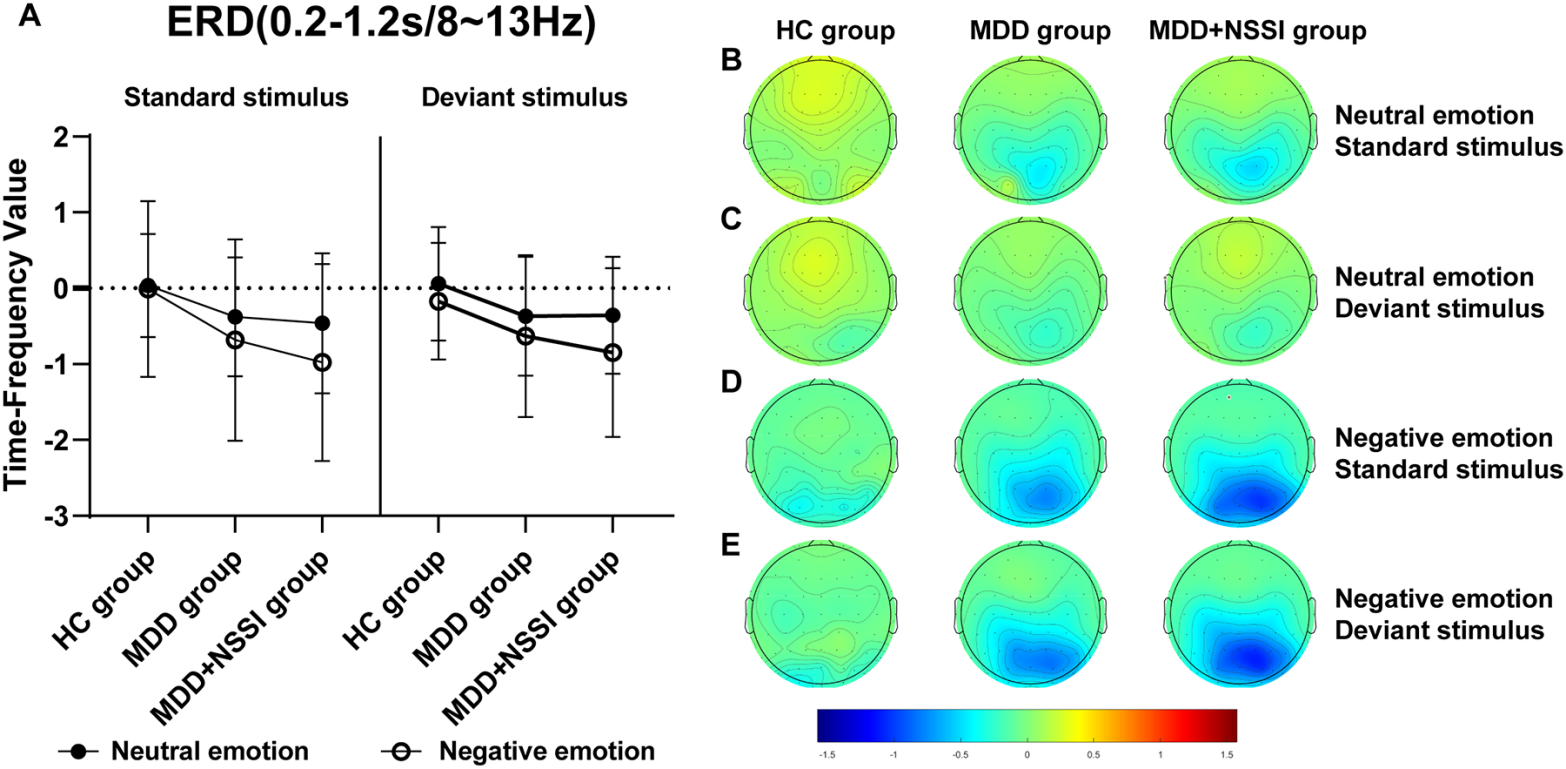
**(A)** Time-frequency values of the time-frequency band of interest (0.2-1.2s/8-13Hz) under different emotional stimulus in HC, MDD and MDD+NSSI groups. **(B)** Time-frequency topography of the time-frequency band of interest (0.2-1.2s/8-13Hz) in HC, MDD and MDD+NSSI groups to neutral emotional standard stimulus. **(C)** Time-frequency topography of the time-frequency band of interest (0.2-1.2s/8-13Hz) in HC, MDD and MDD+NSSI groups to neutral emotional deviant stimulus. **(D)** Time-frequency topography of the interested time frequency band (0.2-1.2s/8-13Hz) in HC, MDD and MDD+NSSI groups under the negative emotional standard stimulus. **(E)** Time-frequency topography of the interested time frequency band (0.2-1.2s/8-13Hz) in HC, MDD and MDD+NSSI groups to the negative emotional deviant stimulus.

**Figure 2** illustrates the time-frequency energy spectra of various frequency bands across time points at the midline electrode under all conditions. Prominent ERS was observed in the Delta and Theta bands at 0-0.6 s on the FCz electrode, while notable ERD occurred in the Theta and Alpha bands at 0.2-1.2 s on the Pz electrode. The findings are summarized in **Table 4** and illustrated in **Figures 5**–**8**.

**Table 4:** Time-frequency values of participants in the HC group, MDD group, and MDD+NSSI group exposed to different emotional cues.

Time-Frequency Values		Emotion main effect *F(p)*	Stimulus main effect *F(p)*	Between-group main effect *F(p)*	Emotion × Stimulus interaction effect *F(p)*	Emotion × Group interaction effect *F(p)*	Stimulus × Group interaction effect *F(p)*	Emotion × Stimulus × Group interaction effect *F(p)*
ERS	0-0.6s/1-4Hz	144.424 (<0.001)	1.550 (0.215)	2.419 (0.043)	1.548 (0.215)	3.920 (0.022)	2.555 (0.081)	0.154 (0.857)
	0-0.6s/5-7Hz	122.383 (<0.001)	3.140 (0.078)	1.049 (0.048)	0.764 (0.384)	3.477 (0.033)	3.054 (0.050)	0.260 (0.771)
ERD	0.2-1.2s/5-7Hz	6.789 (0.010)	0.816 (0.368)	4.221 (0.016)	0.059 (0.808)	2.920 (0.057)	0.296 (0.744)	0.338 (0.714)
	0.2-1.2s/8-13Hz	25.437 (<0.001)	0.200 (0.655)	5.160 (0.007)	0.242 (0.623)	3.246 (0.042)	0.774 (0.463)	0.662 (0.518)

ERS, event‐related synchronization; ERD, event ‐related desynchronization.

##### 3.3.2.1 ERS (FCz electrode, 0-0.6s) Delta Band (1-4 Hz)

For the Delta band, analysis revealed a significant main effect of condition for ERS [F_(1, 148)_= 144.424, *p* < 0.001, η_p_^2^ = 0.494], a significant condition × group interaction [F_(2, 148)_= 3.920, *p* = 0.022, η_p_^2^ = 0.050], and a significant main effect of group [F_(2, 148)_= 2.419, *p* = 0.043, η_p_^2^ = 0.032]. *Post hoc* analysis indicated that the MDD + NSSI group’s Delta band power (0.885 ± 0.655) under negative emotional cues was significantly higher than the MDD group (0.788 ± 0.705, *p* = 0.048) and HC group (0.605 ± 0.479, *p* = 0.036).

##### 3.3.2.2 Theta Band (5-7 Hz)

For the Theta band, a significant main effect of condition for ERS was observed [F_(1, 148)_= 122.383, *p* < 0.001, η_p_^2^ = 0.453], along with a significant condition × group interaction [F_(2, 148)_= 3.477, *p* = 0.033, η_p_^2^ = 0.045] and a significant main effect of group [F_(2, 148)_= 1.049, *p* = 0.048, η_p_^2^ = 0.014]. *Post hoc* analysis revealed that the MDD + NSSI group’s Theta band power (0.936 ± 0.968) under negative emotional cues was significantly greater than the MDD group (0.772 ± 0.992, *p* = 0.031) and HC group (0.727 ± 0.643, *p* = 0.038).

##### 3.3.2.3 ERD (Pz electrode, 0.2-1.2s) Theta band (5-7 Hz)

For the Theta band, analysis revealed a significant main effect of condition for ERD values [F_(1, 148)_= 6.789, *p* = 0.010, η_p_^2^ = 0.044] and a significant main effect of group [F_(2, 148)_= 4.221, *p* = 0.016, η_p_^2^ = 0.054]. *Post hoc* analysis indicated that the MDD + NSSI group exhibited significantly lower Theta band ERD values (-0.475 ± 0.891) under negative emotional cues compared to the MDD group (-0.307 ± 0.753, *p* = 0.038) and the HC group (-0.031 ± 0.474, *p* = 0.006).

##### 3.3.2.4 Alpha band (8-13 Hz)

For the Alpha band, analysis revealed a significant main effect of condition for ERD values [F_(1, 148)_= 25.437, *p* < 0.001, η_p_^2^ = 0.147], a significant condition × group interaction [F_(2, 148)_= 3.246, *p* = 0.042, η_p_^2^ = 0.042], and a significant main effect of group [F_(2, 148)_= 5.160, *p* = 0.007, η_p_^2^ =0.065]. *Post hoc* analysis showed that the MDD + NSSI group’s Alpha band ERD values under negative emotional cues (-0.629 ± 1.066) were significantly lower than those of the MDD group (-0.307 ± 0.753, *p* = 0.047) and the HC group (-0.171 ± 0.770, *p* = 0.006).

## 4 Discussion

This study employed neutral and negative emotional cue tasks to investigate the behavioral and neural responses of depressed adolescents with NSSI to negative emotional stimuli, focusing on their response inhibition capacity. We examined changes in behavioral indicators among depressed adolescents with NSSI, depressed adolescents without NSSI, and healthy controls when exposed to negative emotional stimuli. Additionally, ERP components related to response inhibition were analyzed from both temporal-domain and time-frequency perspectives.

In terms of behavioral performance, there were no significant differences in accuracy cost or reaction time cost across groups, although both showed significant main effects of stimulus type. This indicates that, regardless of the group, participants exhibited lower accuracy and longer RT for deviant stimuli compared to standard stimuli. These findings suggest that deviant stimuli, presented less frequently, are harder to control due to participants’ dominant responses to standard stimuli, leading to increased errors and longer reaction times. Additionally, RT cost showed a significant main effect of emotional condition, indicating that participants across all groups responded faster to negative emotional stimuli than to neutral stimuli. This suggests that negative emotional stimuli capture participants’ attention more effectively than neutral stimuli. However, no significant group differences were observed, consistent with previous findings (50, 51).

At the neural level, depressed adolescents with NSSI exhibited significant N2 components at the FCz electrode and P3 components at the Pz electrode. While no significant group differences were found in N2 latency or amplitude, P3 amplitude showed significant group differences. The MDD+NSSI group displayed significantly higher P3 amplitudes compared to the MDD and HC groups, particularly under negative emotional cues, highlighting increased neural resource allocation for inhibitory control. In previous studies, impaired response inhibition has usually been investigated in terms of both behavioral performance and electrophysiological indicators. In this study, the three groups showed similar behavioral performance, but there were significant intergroup differences in electrophysiological performance. If the behavioral indicators are similar, but one group has a large P3 waveform, it means that this group has to invest more resources in the response inhibition phase to achieve a similar behavioral performance, that is, the response inhibition function of this group is impaired. If a group has an increased P3 waveform and a poor behavioral performance, it means that even if more resources are invested, they are unable to complete the required behavior, indicating that the response inhibition function of this group is more severely impaired. If the amplitude of a group’s P3 waveform is significantly reduced, and the behavioral performance is poor, it means that this group cannot even call on the neural resources related to response inhibition, i.e. the response inhibition function is more and more severely impaired. If the amplitude of a group’s P3 waveform is reduced, but the behavioral indicators are similar or better, then it means that this group is not impaired in response inhibition but has a better response inhibition function. In this study, the MDD+NSSI group had similar behavioral performance compared to the MDD group and the HC group, but the P3 wave amplitude of the MDD+NSSI group was significantly increased. Therefore, these findings suggest impaired inhibitory processes in the MDD+NSSI group, requiring greater cognitive effort to suppress negative emotional stimuli. Consistent with prior findings, the greater salience of substance-related cues for individuals with addiction is associated with enhanced P3 amplitude (52).

Time-frequency analysis revealed the Delta and Theta frequency bands from 0 to 0.6 s at the FCz electrode showed significant ERS, and the Theta and Alpha frequency bands from 0.2 to 1.2 s at the Pz electrode showed significant ERD. Previous studies have shown that Delta and Theta oscillations uniquely contribute to N2 and P3 components, reflecting functional processes related to response inhibition (36). This study also observed that in the time range of 0-0.6s after stimulation, the energy values of the Delta and Theta frequency bands on the FCz electrode of depressed adolescents with NSSI were significantly greater than those of depressed adolescents and healthy adolescents, suggesting that Delta oscillations and Theta oscillations are involved in the N2 component of the response inhibition process. In the time range of 0. 2-1.2s, the energy values of theta and alpha bands on the Pz electrode of depressed adolescents with NSSI were significantly lower than those of depressed adolescents and healthy adolescents, which indicates that theta and alpha oscillations may be involved in the P3 component or even later positive components (such as the LPP) in the response inhibition process. Consistent with previous research, Delta oscillations likely reflect the motivational relevance and salience detection of target stimuli, contributing to ongoing stimulus selection (39). Increased Delta activity indicates greater neural resource allocation for stimulus monitoring and recognition, a process closely associated with the N2 component (40). Theta oscillations may play dual roles: supporting conflict monitoring of emotional stimuli (N2 component) and reflecting response inhibition or processing execution (P3 component) (36). In this study, Theta activity persisted from stimulus onset to 1.2 s, showing increased power at the FCz electrode and decreased power at the Pz electrode. Other researchers have similarly suggested that Theta oscillations are involved in both early and late stages of emotional processing, closely linked to higher-order cognitive functions (40, 53). Additionally, the P3 component has been consistently associated with advanced cognitive processes in previous studies (54). Alpha oscillations, previously linked to emotional processing, show enhanced ERD in posterior regions during unpleasant emotional stimuli (55). This study confirmed that Alpha-ERD at the Pz electrode was significantly stronger in depressed adolescents with NSSI under negative emotional cues compared to both depressed adolescents without NSSI and healthy controls. Moreover, the time of occurrence of Alpha oscillations was later, with a large temporal overlap with the late positive component, suggesting that Alpha oscillations are involved in the fine processing of emotional processing, which is also similar to previous studies (56).

This study also has the following shortcomings: First, NSSI does not only occur in the depression population, but considering the impact of the heterogeneity of the patient population on the study, we only included adolescents with NSSI whose primary diagnosis was MDD. Therefore, the results and conclusions of this study may not be applicable to those adolescents with NSSI but do not meet the MDD diagnosis. Second, the subjects in the MDD+NSSI group were on average younger and more female, which is consistent with epidemiological surveys of NSSI, but unbalanced demographics may still have an impact on our results. Therefore, we included age and sex as covariates in subsequent analyses. Nevertheless, our results still need to be verified in the future using large-scale samples with balanced demographics. Third, the time-frequency analysis relied on a single electrode, which limited the ability to assess the broader developmental trajectory of time-frequency activity. Future research should explore the time-frequency trajectory of NSSI behavior using more comprehensive electrode arrays in depressed adolescents with NSSI.

## 5 Conclusion

This study provides electrophysiological evidence indicating that depressed adolescents with NSSI exhibit increased P3d amplitudes, enhanced Delta/Theta ERS, and heightened Theta/Alpha ERD when exposed to negative emotional stimuli. These findings suggest that impaired response inhibition in depressed adolescents with NSSI may contribute to the occurrence of NSSI behaviors.

## Data Availability

The raw data supporting the conclusions of this article will be made available by the authors, without undue reservation.
